# Canonical and extra‐telomeric functions of telomerase: Implications for healthy ageing conferred by endurance training

**DOI:** 10.1111/acel.13836

**Published:** 2023-04-11

**Authors:** Joshua Denham

**Affiliations:** ^1^ School of Health and Medical Sciences University of Southern Queensland Toowoomba Queensland Australia; ^2^ Centre for Health Research Institute for Resilient Regions Toowoomba Queensland Australia

**Keywords:** epigenetics, exercise, metabolism, oxidative stress, telomeres, *TERT*

## Abstract

Telomerase preserves genomic integrity by maintaining and protecting the telomeres. Seminal findings from 1985 revealed the canonical role of telomerase and motivated investigations into potential therapeutic strategies to combat one of the hallmarks of ageing—telomere attrition. Since then, the field of telomere biology has rapidly expanded, with telomerase serving essential roles in cancer and cell development through its canonical function. However, telomerase also exerts critical extra‐telomeric functions through its protein (telomerase reverse transcriptase, TERT) and RNA components (telomerase RNA component, *TERC*). Telomerase re‐activation or ectopic expression promotes survival and permits unlimited proliferation in tumours and healthy non‐malignant cells. *TERT* gene therapies improve health and lifespan in ageing mice and mouse models of age‐related diseases. The extra‐telomeric functions of telomerase are critical to ageing. These include protection against oxidative stress, orchestration of chromatin modifications and transcription, and regulation of angiogenesis and metabolism (e.g. mitochondrial function and glucose control). Given these biological functions are key adaptations to endurance training and the recent meta‐analytical findings that indicate exercise up‐regulates *TERT* and telomerase, a comprehensive discussion on the implications of the canonical and extra‐telomeric roles of telomerase is warranted. This review highlights the therapeutic benefits of telomerase‐based treatments for idiopathic and chronic diseases that are linked to ageing. Discussion on the canonical and extra‐telomeric roles of telomerase are presented, followed by a detailed summary of the evidence on how exercise influences telomerase. Finally, the potential cell signalling underpinning the exercise‐induced modulation of telomerase are discussed with directions for future research.

## Graphical abstract

Telomerase prevents telomere attrition, which is one of the hallmarks of ageing. The major protein component (telomerase reverse transcriptase, TERT) and RNA template of telomerase also perform extra‐telomeric functions, including transcriptional regulation, angiogenesis, and control of metabolism and mitochondrial function. Considering endurance exercise up‐regulates TERT expression and telomerase activity, regular training could improve healthy biological ageing through the canonical and extra‐telomeric functions of TERT.

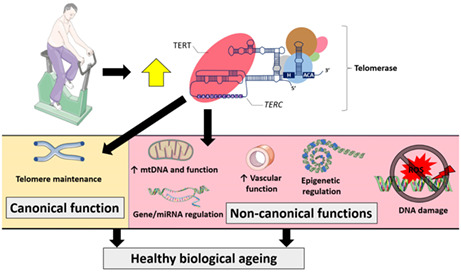

**Abbreviations**
AAV: adeno‐associated virus
ALT: alternative lengthening of telomeres
eNOS: endothelial nitric oxide synthase
GDF11: growth differentiation factor 11
HDAC: histone deacetylase
HUVECs: human umbilical vein endothelial cells
PBMCs: peripheral blood mononuclear cells
RNP: ribonucleoprotein
ROS: reactive oxygen species
*TERC*
: telomerase RNA component
TERT: telomerase reverse transcriptase
VEGF: vascular endothelial growth factor

## 1 INTRODUCTION

Ageing is a complex biological process involving a dynamic network of cellular and molecular activity incompletely understood. Since the early 1960s, it was clear that cells have a finite ability to undergo mitosis before entering a senescent state, although the underlying mechanisms were unclear (Hayflick, 1965; Hayflick & Moorhead, 1961). Cultured mammalian cells enter senescence after a finite number of cell divisions—the Hayflick limit (Shay & Wright, 2000). The failure of DNA polymerase to replicate the entire lagging (‘C’) strand leads to telomere shortening with each round of cell division until senescence ensues, due to the end replication problem (Levy et al., 1992; Wynford‐Thomas & Kipling, 1997). In 1985, telomerase was discovered, and its canonical function of maintaining genomic stability by elongating the telomeres was revealed (Greider & Blackburn, 1985). An RNA template complementary to telomeric sequence and its reverse transcriptase activity underpins the ability of telomerase to extend the terminal DNA, thereby circumventing the end replication problem and replicative senescence (Greider & Blackburn, 1989). The significance of this discovery was acknowledged with the authors receiving the Nobel Prize in Physiology or Medicine in 2009.

## 2 THE CANONICAL ROLE OF TELOMERASE: TELOMERE MAINTENANCE

Telomerase extends the telomeres and attenuates telomere attrition, which is one of the hallmarks of ageing (Lopez‐Otin et al., 2013). Critically, short telomeres cause telomere dysfunction, which triggers DNA damage responses and other factors that promote cellular senescence or apoptosis (d'Adda di Fagagna et al., 2003; Karlseder et al., 1999; Meier et al., 2007). Replicative—critically short telomere‐induced—senescence can occur in rapidly dividing cells (e.g. stem cells and lymphocytes), whereas ROS or genotoxic stress‐induced damage along the telomeres can promote telomere dysfunction and senescence, which is more likely to affect post‐mitotic tissue (e.g. cardiac and skeletal muscle, neurons and fat) (Rossiello et al., 2022). This, in turn, promotes low‐grade chronic inflammation via the senescence‐associated secretory phenotype (De Cecco et al., 2019; Lasry & Ben‐Neriah, 2015) which, in turn, accelerates telomere shortening and biological ageing (Jurk et al., 2014). Telomere dysfunction is implicated in most age‐related diseases (Rossiello et al., 2022), and meta‐analytical findings suggest short telomeres are often observed in patients with chronic diseases compared to healthy controls. These include individuals with obesity (Mundstock, Sarria, et al., 2015), type 2 diabetes (Wang et al., 2016), Alzheimer's disease (Forero et al., 2016) and coronary heart disease (Haycock et al., 2014).

**FIGURE 1:**
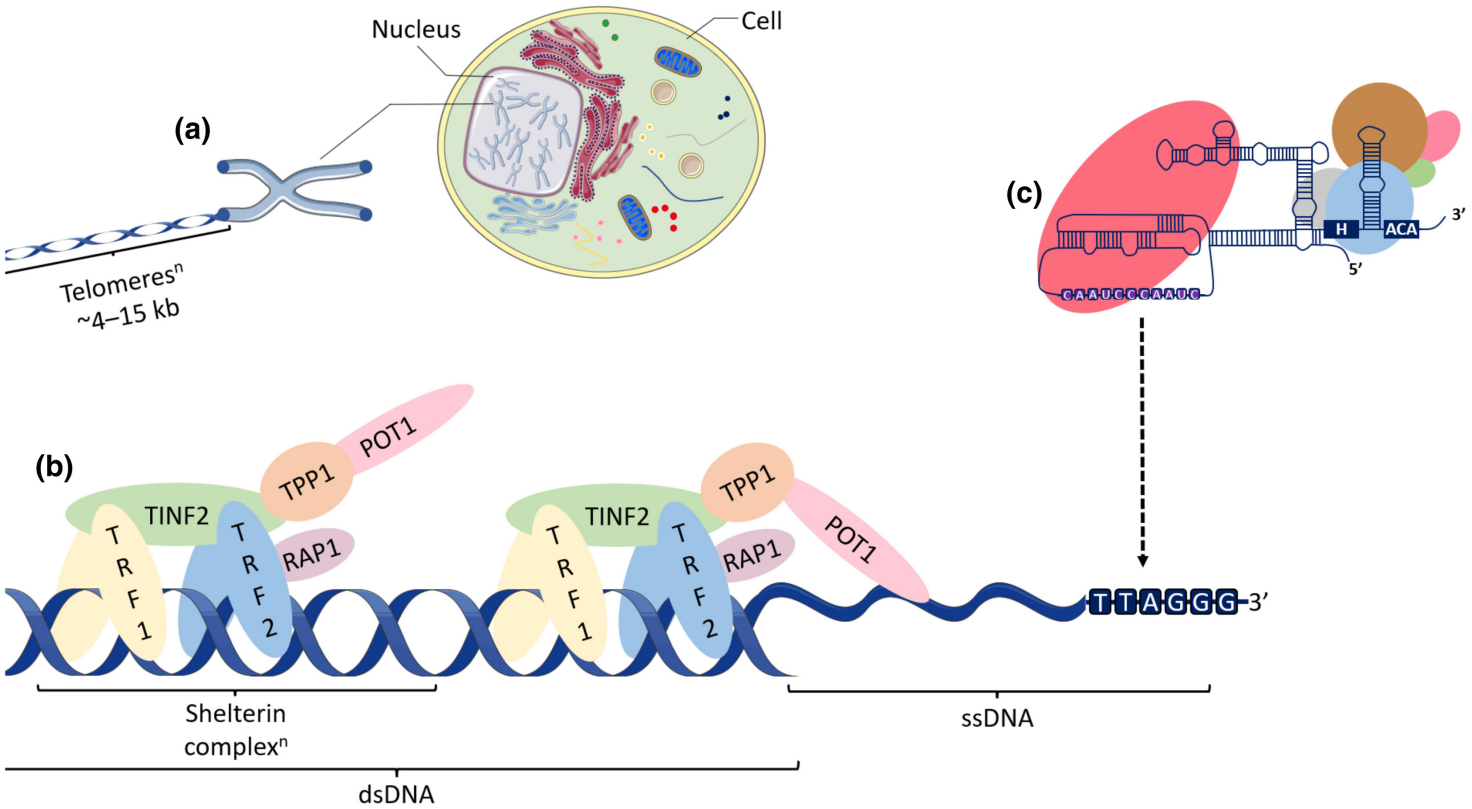
Telomeres, shelterin and the canonical function of telomerase. (a) Telomeres are a repetitive sequence of DNA (5′‐TTAGGG^n^‐3′) that cap the ends of chromosomes and preserve genomic integrity. Average telomere length in humans is typically 4–15 kilobases (kb) long. Given that telomerase is expressed at very low levels in healthy human cells, telomeres shorten with successive cell divisions due to the end replication problem and DNA damage. (b) Shelterin is comprised of six telomere‐repeat binding proteins (telomere repeat binding factor 1 [TRF1], telomeric repeat binding factor 2 [TRF2], TRF1 interacting nuclear factor 2 [TINF2], TRF2 interacting protein [RAP1], ACD shelterin complex subunit and telomerase recruitment factor [TPP1], and protection of telomeres 1 [POT1]) that bind telomeric DNA directly (protein homodimers, TRF1/TRF2) or indirectly (TINF2, TPP1 and RAP1) via TRF1/2. POT1 preferentially binds to single‐stranded telomeric DNA (G‐overhang). Note that this is the open telomere state, as shelterin are responsible for directing other structures at telomeres (closed states) (e.g. T‐ and D‐loops and other formations) (Lim & Cech, 2021; Zinder et al., 2022). (c) Human telomerase is comprised of a catalytic core ribonucleoprotein (RNP) (telomerase reverse transcriptase [TERT] protein and telomerase RNA component [TERC]) and an H/ACA RNP complex with H/ACA ribonucleoproteins: dyskerin (blue), NOP10 (green), NHP2 (pink), GAR1 (grey) and TCAB1 (brown) (Liu et al., 2022; Nguyen et al., 2018). The template region of telomerase interacts with the g‐strand telomeric DNA via the complementary *TERC* sequence (white RNA with purple background) for telomere synthesis. TERT is indicated by the light red oval. Supported by Servier Medical Art.

Telomerase is comprised of ribonucleoprotein (RNP) complexes capable of telomere elongation through its reverse transcription activity (Blackburn et al., 1989; Greider & Blackburn, 1987) (Figure 1). Extensive discussion on the recruitment of telomerase and the mechanism of telomere synthesis has been provided elsewhere (Roake & Artandi, 2020; Wu et al., 2017). Telomeres form specific nucleoprotein complexes at the distal ends of chromosomes facilitated by interactions with six telomere repeat‐binding proteins involved in telomerase recruitment—shelterin (Figure 1). Shelterin are critical to repressing DNA damage response pathways (e.g. ATM/ATR and p53 signalling) (d'Adda di Fagagna et al., 2003; Karlseder et al., 1999), as they form unique telomere structures (e.g. end‐capped telomeres via t‐ and d‐loops) (Lim & Cech, 2021) and compact telomeric chromatin (Bandaria et al., 2016). Shelterin also prevents non‐homologous end joining (Arnoult & Karlseder, 2015; Dimitrova et al., 2008; Sfeir & de Lange, 2012), as excessive telomere shortening and shelterin protein removal from the telomeres causes telomere dysfunction. Comprehensive discussions on shelterin and telomere structures can be found here (de Lange, 2018; Lim & Cech, 2021; Smith et al., 2020). TPP1 coordinates telomerase recruitment and enhances its activity at the telomeres through direct interactions (Liu et al., 2022; Wang et al., 2007). Importantly, TERT is the major protein of telomerase and the rate‐limiting component of the catalytic core ribonucleoprotein. It is heavily regulated and is poorly expressed in most somatic cells. Further, TERT is under epigenetic control by chromatin structural changes caused by telomere shortening; it exhibits epigenetic marks indicative of transcriptional silencing when telomeres are long and transcriptional activation when telomeres are short (Kim et al., 2016). The control and actions of telomerase are the subject of an excellent review (Roake & Artandi, 2020).

Cell and animal models with genetically modified or no telomerase activity have been especially helpful in uncovering the importance of telomerase in telomere maintenance. Population doubling in human fibroblasts causes telomere shortening in vitro (Harley et al., 1990), yet exogenous *Tert* treatment considerably extends their lifespan by maintaining critically short telomeres (Bodnar et al., 1998; Ouellette et al., 2000). Further, increased TERT expression preferentially protects short, dysfunctional telomeres to buffer senescence and avert malignant transformation (Sun et al., 2019). *Terc*‐deficient embryonic stem cells exhibit progressive telomere shortening and impaired growth rates, highlighting the importance of telomerase in mammalian growth and development (Niida et al., 1998). Experiments involving the decrease or complete removal of telomerase activity in several mouse models have uncovered the impact of telomere shortening on biological ageing. Mice lacking the telomerase‐RNA template (*Terc* ^−/−^) or the major catalytic protein (*Tert* ^−/−^) are viable despite the absence of telomerase activity. Whilst maintaining embryonic viability, *Tert* ^−/−^ or *Terc* ^−/−^ mice possess no obvious abnormalities in early generations (Blasco et al., 1997; Yuan et al., 1999). *Terc* ^−/−^ mice become infertile by the G5–G6 and produce less than 50% of the normal progeny by G3 (Herrera et al., 1999). Both *Tert* ^−/−^ and *Terc* ^−/−^ mice display reduced body weights by G3 and progressive telomere shortening with each generation (~4 kb less) (Herrera et al., 1999; Strong et al., 2011) until telomeres are undetectable and end to end fusions occur in late generations (G4–G6) (Blasco, 2002; Blasco et al., 1997; Herrera et al., 1999). That *Tert* ^*−/−*^ and *Terc* ^*−/−*^ mice are not directly comparable due to differences in phenotypes, and some unique extra‐telomeric functions of each telomerase component is worth noting. Survival is significantly reduced in G2 and G3 *Tert* ^−/−^ mice (Strong et al., 2011), whereas this is not observed in *Terc* ^−/−^ mice until the G4s (Herrera et al., 1999). *Terc* ^−/−^ mice are afflicted with immunological defects and other unique phenotypes with ageing beginning in G3s (e.g. atrophy to the spleen, testes and small intestines, hair greying, skin lesions, alopecia, hypertension, blood count abnormalities and other immune deficiencies) (Blasco, 2002; Herrera et al., 1999; Pérez‐Rivero et al., 2006; Rudolph et al., 1999). Similarly, *Tert* ^−/−^ mice suffer intestinal tissue atrophy, immunological abnormalities and depleted tissue renewal capabilities (Strong et al., 2011). However, others reported normal phenotypes in early generation *Tert* ^−/−^ mice (Yuan et al., 1999). Hence, telomere shortening via telomerase interference significantly impacts biological ageing and lifespan.

Considering the crucial role of telomerase in ageing, *Tert*‐based gene therapies were developed and have shown promising results in rodents. Telomerase is tightly restricted in vivo, as high levels are essential for tumour development (Shay & Bacchetti, 1997). However, *Tert* gene therapy enhanced epithelial tissue function, reduced the incidence of tumours and enhanced longevity by 40% in mice resistant to cancer (Tomás‐Loba et al., 2008). This was in contrast to the increased tumour burden in normal mice with long telomeres after *Tert* gene therapy (Artandi et al., 2002), which was exacerbated in mice with a tumour suppressor gene (p53) mutation (Gonzalez‐Suarez et al., 2002). Further, reactivated telomerase activity in mice with dysfunctional telomeres led to telomere elongation and counteracted the age‐associated DNA damage and senescent phenotypes in several tissues (Jaskelioff et al., 2011). Similarly, an adeno‐associated virus (AAV)‐based *Tert* gene therapy improved markers of biological ageing and increased lifespan of 1‐ and 2‐year‐old mice by 24% and 13%, respectively, without increasing tumour incidence (Bernardes de Jesus et al., 2012). Intranasal delivery of *Tert* gene therapy via a cytomegalovirus has demonstrated comparable results to injectable *Tert*, such that telomere shortening, and other ageing markers were attenuated in treated mice (Jaijyan et al., 2022). Similar results were observed after AAV‐based *Trf1* gene therapy in mice (Derevyanko et al., 2017), indicating that gene therapy by increasing a component of shelterin or telomerase improves health and lifespan. Therefore, the canonical role of telomerase is to maintain the telomeres, prevent telomere dysfunction and biological ageing.

## 3 TELOMERES AND TELOMERASE IN RARE AND AGE‐RELATED CHRONIC DISEASES

Telomere syndromes are a spectrum of rare diseases with common defining traits of accelerated biological ageing, reduced lifespan and short, dysfunctional telomeres (Armanios & Blackburn, 2012). Accelerated telomere attrition in the telomere syndromes is due to rare mutations in genes that control telomere integrity, including protein components of telomerase. Dyskeratosis congenita was the first rare disease classified as a telomere syndrome. Mutations in the gene coding for dyskerin (dyskeratosis congenita 1 [*DKC1*]) were revealed and established as a vital protein component of telomerase (Mitchell et al., 1999) (Figure 1). Consequently, individuals with dyskeratosis congenita, as well those with a severe form of the disease, Hoyeraal Hreidarsson syndrome, possess short telomeres, exhibit physical signs of accelerated ageing and die prematurely (Alter et al., 2012; Bessler et al., 2010; Mitchell et al., 1999). Telomere syndromes with mutations in other genes coding essential telomerase proteins are now established (e.g. *NOP10*, *NHP2*, *GAR1* and *TCAB1*) (Mangaonkar & Patnaik, 2018; Mitchell et al., 1999). Interestingly, mouse models lacking telomerase components (*Tert*/*Terc*) recapitulate the accelerated ageing phenotypes (e.g. premature greying, ineffective haematopoiesis, impaired immunity and fibrosis) observed in patients with telomere syndromes (Armanios & Blackburn, 2012). Telomere syndromes are serious and debilitating rare diseases, yet telomerase and telomere shortening are also implicated in common age‐related disease. Telomerase therapy may have the potential to treat not only the telomere syndromes, but also common age‐related diseases.

Telomerase‐mediated telomere maintenance and protection against telomere dysfunction are crucial for the prevention of many age‐related chronic diseases and symptoms, such as atherosclerosis (Chen et al., 2014; Samani et al., 2001; Toupance et al., 2017), kidney (Saraswati et al., 2021) and pulmonary (Liu et al., 2019) fibrosis, glucose intolerance (Guo et al., 2011) and insulin resistance (Minamino et al., 2009), type 2 diabetes (Cheng et al., 2021; Sampson et al., 2006), cardiomyopathy (Leri et al., 2003), amyotrophic lateral sclerosis (Eitan et al., 2012) and Alzheimer's disease (Shim et al., 2021; Spilsbury et al., 2015). In mouse models, *Tert* gene therapy seems to be an effective strategy to combat age‐related diseases and extend health span. For instance, *Tert* gene therapy improves healing and the function of tissues that were once thought of as irreparable, such as myocardial infarction induced cardiac damage (Bär et al., 2014) and pulmonary fibrosis (Povedano et al., 2018). *Tert* gene therapy also attenuates the impact of neurodegeneration (Whittemore et al., 2019) and aplastic anaemia (Bär et al., 2016), both of which are linked to telomeres dysfunction. Thus, *Tert* gene therapy shows promise for combatting age‐related diseases and ageing in humans. Extensive discussion on telomere‐based therapies can be found here (Chakravarti et al., 2021; Martinez & Blasco, 2017; Yeh et al., 2019).

Most neoplasms have abnormally and critically short telomeres that rely on their high telomerase activity to support uncontrolled proliferation (Shay & Bacchetti, 1997). Only 10–15% of tumours employ telomerase‐independent alternative lengthening of telomeres (ALT) (Cesare & Reddel, 2010; Heaphy et al., 2011; Pickett & Reddel, 2015). That telomere length (replicative senescence), telomerase regulation and cancer risk are intricately linked should not be understated. However, a comprehensive discussion on telomerase, ageing and cancer risk is outside the scope of this review; the reader is referred elsewhere for in‐depth reviews (Lansdorp, 2022; Martinez & Blasco, 2010; Shay, 2016; Shay & Wright, 2019). A key strategy of TERT regulation is achieved via its promoter mutations. In 2013, two groups reported TERT promoter mutations upstream of the transcription start site, which significantly increased its transcription through transcription factor binding (e.g. ETS) (Horn et al., 2013; Huang et al., 2013). The TERT promotor mutations are now acknowledged as the most common non‐coding mutations in tumours (Bell et al., 2016; Heidenreich & Kumar, 2017).

Telomerase activity may have co‐evolved with body mass, such that telomerase activity is lacking or at minimal levels in large mammals (e.g. humans) compared to smaller animals to reduce their risk of cancer (Gomes et al., 2011; Gorbunova & Seluanov, 2009). Rodents possess longer telomeres that undergo faster telomere attrition than human cells, yet replicative senescence is primarily limited to proliferating human somatic cells compared to extrinsic factors in rodents (Itahana et al., 2004). The reader should be mindful of these considerations when interpreting findings from rodents. Despite the considerable attention telomerase has attracted as a target for novel anti‐cancer therapies, none have survived extensive scientific enquiry in stage I–III human clinical trials. Therapeutic treatments that target telomerase in tumours have been challenging (Gao & Pickett, 2022; Guterres & Villanueva, 2020), as some healthy somatic cells express very low levels of telomerase activity (e.g. lymphocytes) and key components of telomerase (e.g. TERT/TERC) are ubiquitously expressed. Nonetheless, CRISPR (Li, Li, et al., 2020), immunotherapies and small molecule inhibitors remain exciting possibilities for future anti‐cancer therapies (Guterres & Villanueva, 2020). The precision of TERT‐based anti‐cancer therapies will be crucial to overcome off‐target side‐effects, especially given the extra‐telomeric functions of telomerase.

## 4 EXTRA‐TELOMERIC FUNCTIONS OF TELOMERASE

**FIGURE 2:**
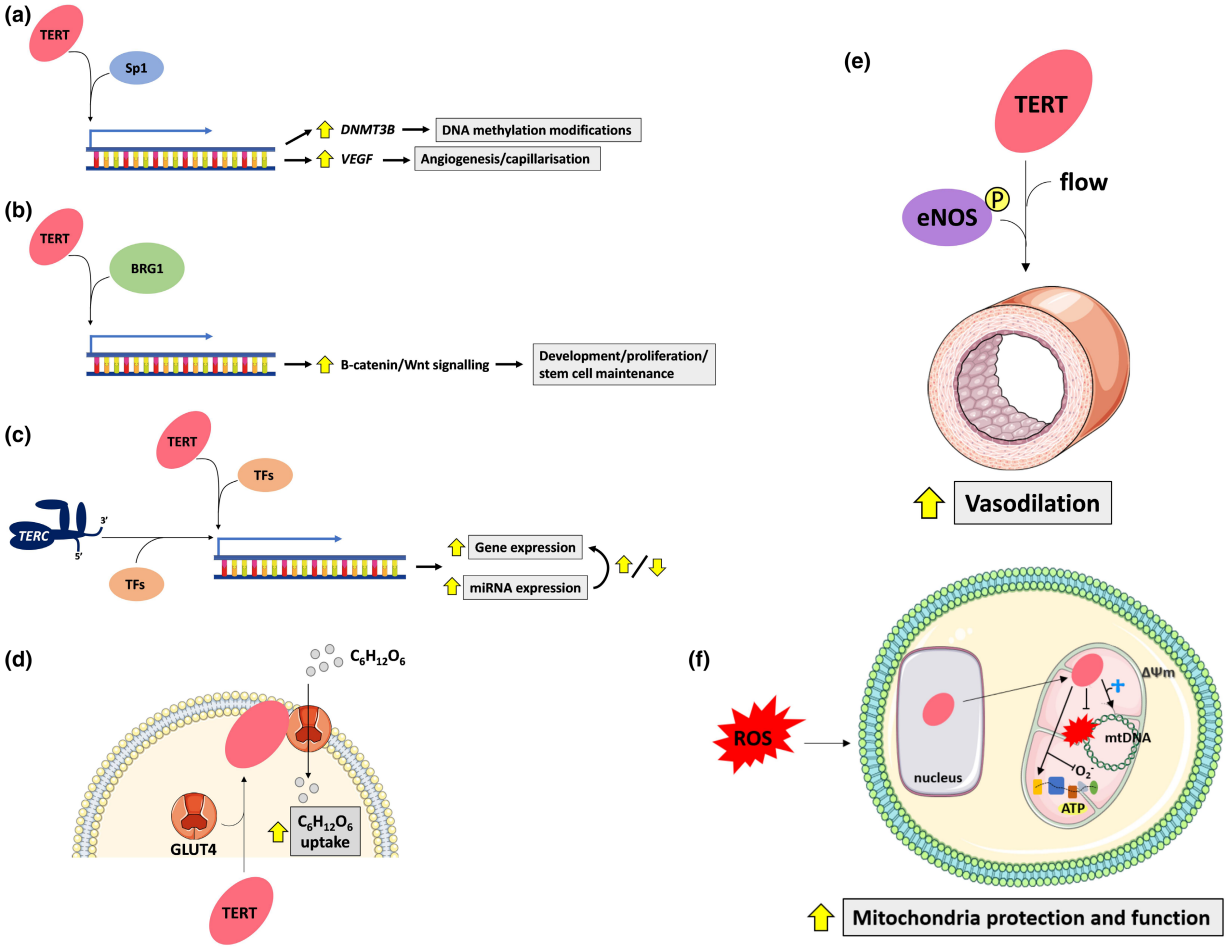
Summary of the key non‐canonical functions of telomerase relevant to endurance training adaptations. (a) TERT binds to Sp1 and transcribes its target genes *DNMT3B* (Yu et al., 2018) and *VEGF* (Liu et al., 2016) thereby contributing to de novo DNA methylation changes and angiogenesis, respectively. This appears to be a telomere length‐independent non‐canonical function of TERT, as catalytically inert TERT exerts identical functions (Liu et al., 2016). (b) TERT interacts with BRG1, which targets genes involved in the β‐catenin/Wnt signalling pathway to regulate development and stem cell maintenance. (c) TERT and TERC appear to independently regulate gene and microRNA (miRNA) expression likely through cooperation with other transcription factors (TFs) to controls genes (e.g. those regulating cell senescence). (d) TERT is shuttled to the plasma membrane upon insulin treatment. There, it colocalises with glucose (C_6_H_12_O_6_) transporters (1, 4 and 12) and increases glucose uptake in skeletal muscle (Shaheen et al., 2014). (e) Increased *TERT* expression via its transcriptional activator, AGS‐499, increases flow‐mediated vasodilation in HUVECs from coronary artery disease patients via enhancing endothelial nitric oxide synthase (eNOS) signalling (Beyer et al., 2016). (f) TERT is shuttled from the nucleus into the mitochondria in the presence of elevated reactive oxygen species (ROS). Inside the mitochondria, TERT improves mitochondrial function and protects mitochondrial DNA (mtDNA) from ROS‐induced damage. It also acts as a reverse transcriptase with mitochondrial transfer RNAs, increases oxygen flux in the electron transport chain and enhances membrane potential (∆Ψ m). Supported by Servier Medical Art.

Telomerase components regulate biological processes in addition to telomere synthesis. Of note, it is difficult to distinguish telomere length dependent compared to telomere independent roles of telomerase. If it is quantified, the average telomere length is typically reported. It is, however, possible that TERT could lengthen critically short (dysfunctional) telomeres on specific chromosomes to circumvent replicative senescence, as well as exert its extra‐telomeric functions. This would seem unlikely when catalytically inert TERT or TERC are used in experiments, as they cannot lengthen telomeres. It should also be noted that the mechanisms by which TERT exerts its non‐canonical functions remain incompletely understood in most circumstances. This next section focuses on the non‐canonical functions of telomerase with a focus on those relevant to physiological adaptations to endurance training (summarised in Figure 2).

### 4.1 Angiogenesis

TERT enhances angiogenesis in endothelial cells, skeletal muscle and malignant tissue via vascular endothelial growth factor (VEGF), endothelial nitric oxide synthase (eNOS) and ERK1/2 signalling pathways (Hoier & Hellsten, 2014; Li, Qian, et al., 2020; McAllister et al., 2008; Suvorava & Cortese‐Krott, 2018). For example, ectopic TERT expression increases tube‐like structure formation in cultured human umbilical vein endothelial cells (HUVECs), by interacting with Sp1 transcription factor, binding to the *VEGF* promoter and up‐regulating its expression (Liu et al., 2016). The angiogenesis relied on TERT, but not its catalytic activity, as an hTERT mutant (lacking telomere synthesis capabilities) stimulated angiogenesis, which indicated a telomere length‐independent effect (Liu et al., 2016). In a clinical study, TERT up‐regulation facilitated growth differentiation factor 11 (GDF11)‐mediated restoration of VEGFR2^+^/CD133^+^ cells isolated from older adult patients who had suffered myocardial infarctions via eNOS and SMAD 2/3 signalling (Zhao et al., 2019). Conversely, vascular function and angiogenesis were impaired in TERT‐depleted endothelial progenitor cells in vitro (Zhao et al., 2019), consistent with other findings (Liu et al., 2016). Using a mouse model of hindlimb ischemia (surgical removal of femoral artery), adenovirus vector treatment with human *VEGF*_*165*_ enhanced skeletal muscle TERT protein expression, telomerase activity, capillarisation and survival in Wistar rats (Zaccagnini et al., 2005). *TERT* transfer alone promoted capillarisation and survival, although to a lesser extent than *VEGF*_*165*_ treatment (Zaccagnini et al., 2005). Moreover, dominant negative *TERT* impaired the VEGF‐induced angiogenesis in skeletal muscle and HUVECs, which emphasised that telomerase activity was required (Zaccagnini et al., 2005). Notwithstanding conflicting reports on whether a functional enzyme is required, together, they indicate TERT induces angiogenesis in healthy tissue.

A role for TERT in angiogenesis was first described in tumours. Tumours sustain growth by up‐regulating angiogenesis, hijacking healthy vascular structures and through the recruitment of endothelial cells (Junttila & de Sauvage, 2013). High *TERT* expression was first identified in vascular endothelial cells isolated from astrocytic tumours which correlated with the severity, such that glioblastoma samples expressed significantly higher *TERT* compared to low‐grade astrocytomas (Pallini et al., 2001). Since these initial findings, TERT now has a well‐established role in tumour growth and development. Using Lewis Lung carcinoma xenograft experiments, *Tert* ^−/−^ mice exhibited reduced tumour growth and micro‐vessel density compared to wild types (Liu et al., 2016), highlighting tumour progression and capillarisation relies on TERT. Others have demonstrated that VEGF‐mediated *TERT* mRNA and telomerase up‐regulation in human ovarian cancerous cell (PA‐1 and SW626) via ERK1/2 signalling and Sp1 (Bermudez et al., 2007), suggesting a possible positive feedback loop since TERT binds to Sp1 and increases *VEGF* expression in HUVECs (N. Liu et al., 2016). Thus, TERT not only maintains neoplasm survival through its canonical function, but it also appears to sustain tumour progression by supporting angiogenesis.

### 4.2 Metabolism

Telomerase seems to be linked to metabolism through both telomere‐length dependent and extra‐telomeric effects. The most compelling evidence implicating telomerase in metabolic functions stems from *Tert* ^−/−^ and *Terc* ^−/−^ mouse experiments. Both Generation 2 (G2) *Terc* ^−/−^ and G4 *Tert* ^−/−^ mice exhibit marked reductions in *Pgc1α*/*β* and downstream metabolic genes, and enforced expression of *Tert* or *Pgc1α* restored the dysregulated transcriptional profile in the heart and liver (Sahin et al., 2011). Mitochondrial copy number, mitochondrial respiration and ATP content were reduced in G1 and G4 *Tert* ^−/−^ as well as G2 *Terc* ^−/−^ mice (Sahin et al., 2011). The metabolic dysfunction also impaired cardiac function and altered gluconeogenesis in G4 *Tert* ^−/−^ mice (Sahin et al., 2011). Determining whether the metabolic compromise in G4 *Tert* ^−/−^ mice is due to telomere dysfunction or the extra‐telomeric functions of TERT (or both) is challenging. That the metabolic compromise is much worse in G4 versus G1 *Tert* ^−/−^ mice (Sahin et al., 2011) may imply that the effects are partly mediated by telomere dysfunction caused by short telomeres. This is supported by evidence from mice genetically engineered to possess much longer (hyper‐long) telomeres compared to controls, as they exhibit less adiposity and LDL cholesterol, superior insulin and glucose sensitivity, increased mitochondrial DNA (mtDNA) and function, and live longer than controls with shorter telomeres (Munoz‐Lorente et al., 2019). Importantly, both studies emphasised links between telomere dynamics, mitochondria and metabolic function, and lifespan. C2C12 cells transfected with a TERT plasmid also exhibited improved glucose uptake and small interfering RNA (siRNA)‐induced knockdown of *TERT* reduces 2‐deoxyglucose uptake in vitro (Shaheen et al., 2014). That TERT was associated with glucose transporters (GLUT1, 4 and 12) and colocalised near the plasma membrane upon insulin treatment in C2C12 cells suggested glucose uptake was mediated by the glucose transporters, yet independent of insulin signalling regulators (PI3K and mTOR) (Shaheen et al., 2014). Regardless of the potential off‐target effects of siRNAs, these data support a role of TERT in glucose control in human skeletal muscle in the absence of telomere dysfunction (normal telomere lengths).

Mice already vulnerable to oxidative stress undergo rapid vascular ageing after *Pgc1α* knockout. Significant vascular cellular senescence occurs with ageing in *Pgc1α* ^*−/−*^ *ApoE* ^*−/−*^ mice, such that they exhibited reduced TERT protein and telomerase activity, as well as increased oxidative DNA damage and short telomeres in the aorta compared to their *Pgc1α* ^*+/+*^ *ApoE* ^*−/−*^ counterparts (Xiong et al., 2015). Furthermore, TERT expression was significantly increased whilst p53 was decreased in rat aortic smooth muscle cells after enforced expression of PGC1α in a dose‐dependent manner (Xiong et al., 2015). Thus, their appears to be a positive feedback loop between PGC1α and TERT. Although it is difficult to determine telomere length‐independent effects of TERT, the positive feedback loop with PGC1α is of interest because telomerase activity is low in healthy human somatic cells, yet *TERT* and *TERC* are ubiquitously expressed. These findings also highlight the complex relationship between telomere length (or telomere dysfunction), telomerase and metabolism, which is undoubtedly supported by the extra‐telomeric effects of TERT in the mitochondria.

### 4.3 Mitochondrial protection and function

TERT not only performs its canonical functions in the nucleus, rather it is also detectable in the mitochondria (including telomerase activity) where it protects against oxidative stress and mtDNA damage. Given that telomeres are absent from the circular mitochondrial genome, the localisation of TERT and telomerase activity in the mitochondria was an exciting finding. Elevated reactive oxygen species (ROS) triggers the nuclear export of TERT from the nucleus into the cytosol (Haendeler et al., 2004; Haendeler, Hoffmann, Brandes, et al., 2003), later identified as the mitochondria (Ahmed et al., 2008; Santos et al., 2004). In fact, approximately 10–20% of TERT seems to reside within the mitochondria under normal physiological conditions (Haendeler et al., 2009; Sharma et al., 2012), whereas it increases to ~80% in the presence of high ROS. Mitochondrial TERT, under high oxidative stress (i.e. hyperoxia or H_2_O_2_), protects mtDNA from lesions and superoxide generation, and boosts mitochondrial membrane potential in cultured fibroblasts (Ahmed et al., 2008). However, different cellular stressors have unique effects in particular somatic cells. Whilst irradiation increases mitochondrial TERT in Purkinje neurons (Eitan et al., 2016), it was suppressed in human breast cancer (MCF‐7) cells (Miao et al., 2016). It is postulated that mitochondrial located TERT protects against ROS‐induced damage to the nucleus in cancer cells, thereby enhancing apoptosis resistance (Singhapol et al., 2013). This presents a challenge for telomerase‐based anti‐cancer therapies, as unwelcome off‐target effects of healthy tissues are possible.

In the mitochondria, TERT acts as a TERC‐independent reverse transcriptase, as it utilises tRNAs as templates and may facilitate mtDNA replication or repair (Sharma et al., 2012). Since mitochondrial biogenesis and function are impaired in many age‐related diseases, this was a particularly interesting finding. In the absence of TERT, the mitochondria exhibit marked mtDNA damage, elevated ROS generation and perturbed ultrastructure indicative of mitochondrial dysfunction (Sharma et al., 2012). Therefore, TERT protects the mitochondria from elevated ROS and subsequent ROS‐induced damage. This function appears to be one of the many ways tumours promote cell survival via TERT (Indran et al., 2011; Pestana et al., 2017), aside from its canonical role of maintaining critically short telomeres.

TERT protected mtDNA from UV damage and H_2_O_2_‐induced apoptosis and enhanced mitochondrial respiration in lung fibroblasts of second‐generation *Tert* ^−/−^ mice and HUVECs, respectively (Haendeler et al., 2009). Furthermore, cardiac mitochondrial respiration was impaired in *Tert* ^−/−^ mice compared to wild types, whereas liver mitochondrial respiration was preserved possibly because more quiescent cells or those that possess higher mitochondrial respiration may rely on TERT for protection (Haendeler et al., 2009). Regarding functional effects ex vivo, telomerase transcriptional activation via AGS 499 restored flow‐mediated vasodilation through nitric oxide and ATP generation in vasculature from patients with coronary artery disease, possibly due to the suppression of ROS (Beyer et al., 2016). Notably, the samples from patients exhibited low TERT protein levels yet possessed normal cardiac (mean) telomere lengths (Beyer et al., 2016). These findings suggest an extra‐telomeric and possibly a telomere length‐independent role of telomerase in vascular function. Together, these findings support a non‐canonical role of TERT in the protection and function of mitochondria.

### 4.4 Gene expression regulation

TERT expression is linked to transcriptional regulation, chromatin structural alterations and post‐transcriptional control via small non‐coding RNAs. Using knock‐in experiments and fluorescence‐activated cell sorting, mouse hepatocytes that expressed either relatively low or high *Tert* expression and telomerase activity were isolated and underwent RNA sequencing (Lin et al., 2018). Notably, 3172 genes were differentially expressed between high and low *Tert* expressing hepatocytes, with key differences in cell cycle and metabolic pathways (Lin et al., 2018). For example, hepatocytes with high *Tert* expression demonstrated up‐regulated gene pathways involving Ras protein signal transduction, MAPK pathway and mitotic spindle, whereas mitochondrion, electron carrier activity and glycolysis/gluconeogenesis signalling were downregulated relative to the low *Tert* hepatocytes (Lin et al., 2018). Similarly, acute withdrawal of TERT in transgenic mice caused dynamic changes in 418 and 255 down and up‐regulated genes, respectively, in epithelial cells, especially in genes enriched for signal transduction, development and cell to cell signalling (Choi et al., 2008). These findings are supported by human in vitro experiments on mammary epithelial cells, whereby ectopic expression of *TERT* also elicited genome‐wide transcriptional changes, which immortalised them in the absence of telomere dysfunction (Smith et al., 2003). Even in cells lacking telomere dysfunction, it appears that TERT modulates genome‐wide transcription in numerous tissues in genes controlling growth, development and metabolism.

Conversely, telomere dysfunction caused by *Tert* ^*−/−*^ or *Terc* ^*−/−*^ significantly deregulates transcriptional landscapes. G4 *Tert* ^−/−^ mice exhibit differentially expressed probes in hematopoietic stem cells (*n* = 280), heart (1544) and the liver (1357) compared to wild‐type controls in genes related to oxidative phosphorylation, oxidative stress and gluconeogenesis (Sahin et al., 2011); possibly a downstream effect of the mitochondrial compromise associated with dysfunction telomeres. Interestingly, most probes appeared to be downregulated in tissue from *Tert* ^−/−^ mice (Sahin et al., 2011), whereas increased *Tert* expression is associated with predominantly up‐regulated transcripts in mouse liver (Lin et al., 2018). Genome‐wide transcriptional changes were also observed in G2 *Terc* ^−/−^ mouse liver (*n* = 1832) and heart (*n* = 1754) (Sahin et al., 2011) with normal telomeres. These data indicate that the deletion of either the catalytic protein or the RNA template of telomerase modulates the transcriptome. Similarly, modest and complete *Tert* deficiency differentially regulates the expression of numerous genes involved in amyloid precursor proteinmetabolic processes in the brain of *Tert* ^−/−^ and *Tert* ^+/−^ mice, respectively (Shim et al., 2021). Moreover, neuronal TERT induction significantly modulates the transcriptome of human and mouse Alzheimer's disease neurons (Shim et al., 2021). Others, however, failed to find significant transcriptional changes in liver from G1 *Tert* ^−/−^ nor G1 *Terc* ^−/−^ mice with long telomeres compared to wild types (Vidal‐Cardenas & Greider, 2010), indicating that gene expression changes may not be independent of telomere length or tissue‐specific regulation.

Although less studied than TERT, *TERC* also appears to regulate genome‐wide gene expression. Its capacity to influence gene activity seems to be telomere length independent, as TERT is the major rate‐limiting component of telomerase activity. *TERC* may act in concert with transcription factors. For instance, short hairpin RNA (shRNA) knockdown of *TERC* downregulated genes involved in myelopoiesis (CSF2, CSF3, SPI1) in human HL60 and U937 cells (neutrophil and monocyte progenitors) that relied on the recruitment of RNA polymerase II (García‐Castillo et al., 2021). Additionally, TERC is imported into the mitochondria, truncated to a shorter version (TERC‐53) and shuttled back into the cytosol where it regulates gene expression and cellular senescence independent of telomerase activity and telomere length (Zheng et al., 2019). Over expression of TERC‐53 in HEK293 cells led to 87 differentially expressed genes, including increased *SIRT1* expression, and others enriched for response to calcium ions, DNA replication proofreading and cell adhesion (Zheng et al., 2019). TERC may serve other functions, given that it is ubiquitously expressed, as well as the regulatory power and multiple targets of other long non‐coding RNAs. Since TERC interacts with the argonaute 2 protein and enhances telomerase activity (Laudadio et al., 2019), it could also inhibit target mRNA translation. The argonaute protein's primary responsibility is to guide small RNAs to complementary mRNA (target) sequences (e.g. siRNAs and microRNAs). Other regulatory functions of TERC have been recently discussed (Gala & Khattar, 2021).

TERT can influence protein abundance through post‐transcriptional regulation, another mechanism controlling gene expression. It was discovered that TERT interacts with RNA other than TERC (e.g. RNA component of mitochondrial RNA processing endoribonuclease [*RMRP*]) and subsequently serves as an RNA polymerase to produce double‐stranded RNAs in cancer cell lines (HeLa, 293T and MCF7) (Maida et al., 2009). The double‐stranded RNAs are processed by DICER1 into siRNAs (Maida et al., 2009) that suppress protein translation by degrading their target mRNAs in a sequence‐specific manner (Hu et al., 2020; McManus & Sharp, 2002). As for the genome‐wide gene expression changes in tissue with altered *Tert* (Lin et al., 2018; Sahin et al., 2011), primary and mature miRNAs are also differentially regulated by *Tert* expression (Lassmann et al., 2015). For example, 12 miRNAs were downregulated after siRNA‐*Tert* suppression in human leukemia (THP‐1) cells (Lassmann et al., 2015). Furthermore, two different siRNAs targeting *Tert* downregulated 77 miRNAs, whereas only nine were up‐regulated in HeLa cells (Lassmann et al., 2015). Cancers have widespread transcriptional changes compared to non‐transformed healthy tissue, yet the influence of TERT on miRNA expression is not limited to neoplasms. SiRNA knockdown of *Tert* significantly reduced the miR‐21, ‐29a and ‐208a (2.7–3.6‐fold) in neonatal cardiac ventricles from Wistar rats (Drevytska et al., 2014). Given that mature miRNAs were modulated and no changes were observed in the primary miRNAs nor miRNA processing proteins at the mRNA level (Drevytska et al., 2014), *Tert* knockdown may regulate miRNA expression indirectly rather than at the transcriptional level or by influencing miRNA maturation. Despite the potential off‐site effects of siRNAs (Hu et al., 2020; Jackson & Linsley, 2010; McManus & Sharp, 2002), the available evidence suggests modulating TERT controls transcriptional reprogramming and may influence mature miRNA abundance.

### 4.5 Epigenetic control

Chromatin conformational changes occur via numerous epigenetic modifications, such as DNA methylation and histone modifications. The latter appears to involve TERT, as its suppression by a shRNA in human fibroblasts impaired DNA damage response to ionising radiation and decreased histone (H) 3 lysine (K) 9 di‐methylation and H4K12 acetylation, and increased H3 K9 acetylation (Masutomi et al., 2005). Three DNA methyltransferases are responsible for DNA methylation maintenance (DNMT1) and de novo modifications to the DNA methylome (DNMT3A/B). TERT promotes the transcription of *DNMT3B* through interactions with Sp1 (Yu et al., 2018). Inhibiting TERT with an siRNA decreases tumour suppressor gene (*PTEN*) promoter and genome‐wide DNA methylation in hepatocellular carcinoma cell lines (Yu et al., 2018). TERT also cooperates with the chromatin remodeller, BRG1. BRG1 is an ATP‐dependent chromatin remodeller that utilises energy from ATP hydrolysis to modify histone‐DNA contacts (e.g. histone deacetylase [HDAC] regulation), alter chromatin confirmation and regulate gene expression (de la Serna et al., 2006; Trotter & Archer, 2008). TERT interacts with BRG1 and modulates Wnt/β‐catenin signalling (Park et al., 2009). Considering that β‐catenin regulates *Tert* expression, there seems to be positive feedback loop between TERT and β‐catenin (Hoffmeyer et al., 2012). Therefore, TERT is involved in the regulation of the epigenome by influencing key proteins involved in histone modifications and DNA methylation changes.

## 5 EXERCISE AND TELOMERASE IN HEALTHY AGEING: KEY POINTS AND OPPORTUNITIES

The extra‐telomeric and canonical functions of telomerase offer plausible explanations on how regular exercise training promotes healthy biological ageing. A recent meta‐analysis involving exercise studies in humanleukocytes and rodent tissues indicated both a single bout and chronic exercise training increases *TERT* gene expression, protein content and telomerase activity (Denham & Sellami, 2021). Furthermore, endurance athletes exhibited higher *TERT* expression and telomerase activity (Denham & Sellami, 2021), which could account for reports that athletes generally possess longer leukocyte telomeres relative to physically inactive individuals (Denham et al., 2013; Denham, O'Brien, Prestes, et al., 2016; LaRocca et al., 2010; Werner et al., 2009). The meta‐analytical findings could account for observations from epidemiological studies that indicated a positive or inverted ‘U' relationship between physical activity and telomere length, mainly in leukocytes (Denham, O'Brien, & Charchar, 2016; Ludlow et al., 2008; Mundstock, Zatti, et al., 2015; Valente et al., 2021). Leukocyte telomere length is the most widely studied somatic cell not only due to the ease of collection, relatively painless procedure and high DNA yields, but because it reflects telomere length in haematopoietic stem cells and correlates with other tissues (e.g. skeletal muscle, skin, leukocyte subsets and fat) (Daniali et al., 2013; Kimura et al., 2010).

**FIGURE 3:**
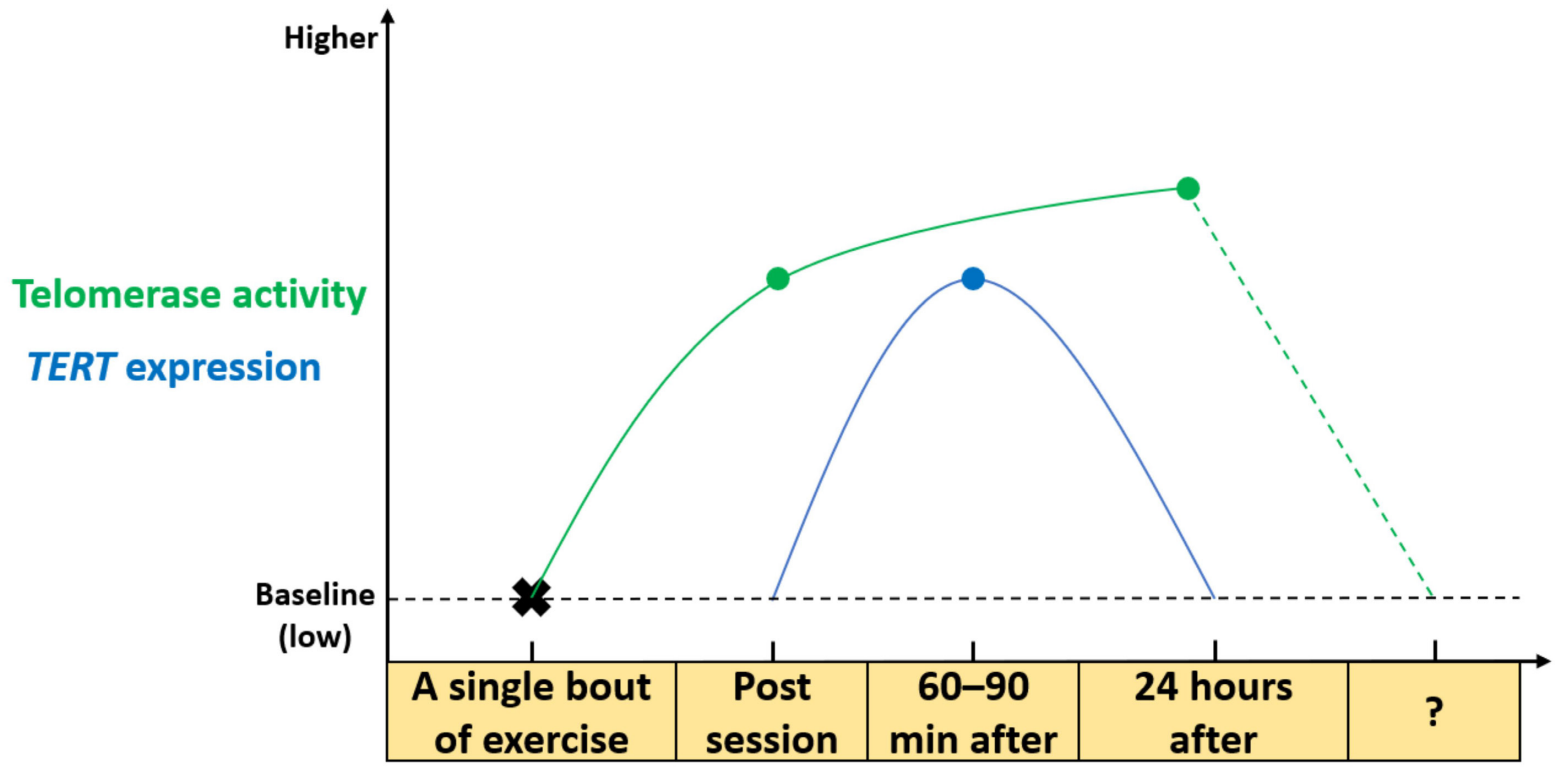
Time course of endurance exercise‐induced changes in *TERT* expression and telomerase activity. A single bout of endurance exercise increases TERT gene expression 60–90 min after the cessation of the exercise session. Telomerase activity is, however, up‐regulated immediately after training and remains elevated—slightly higher than post‐session values—24 h after.

**FIGURE 4:**
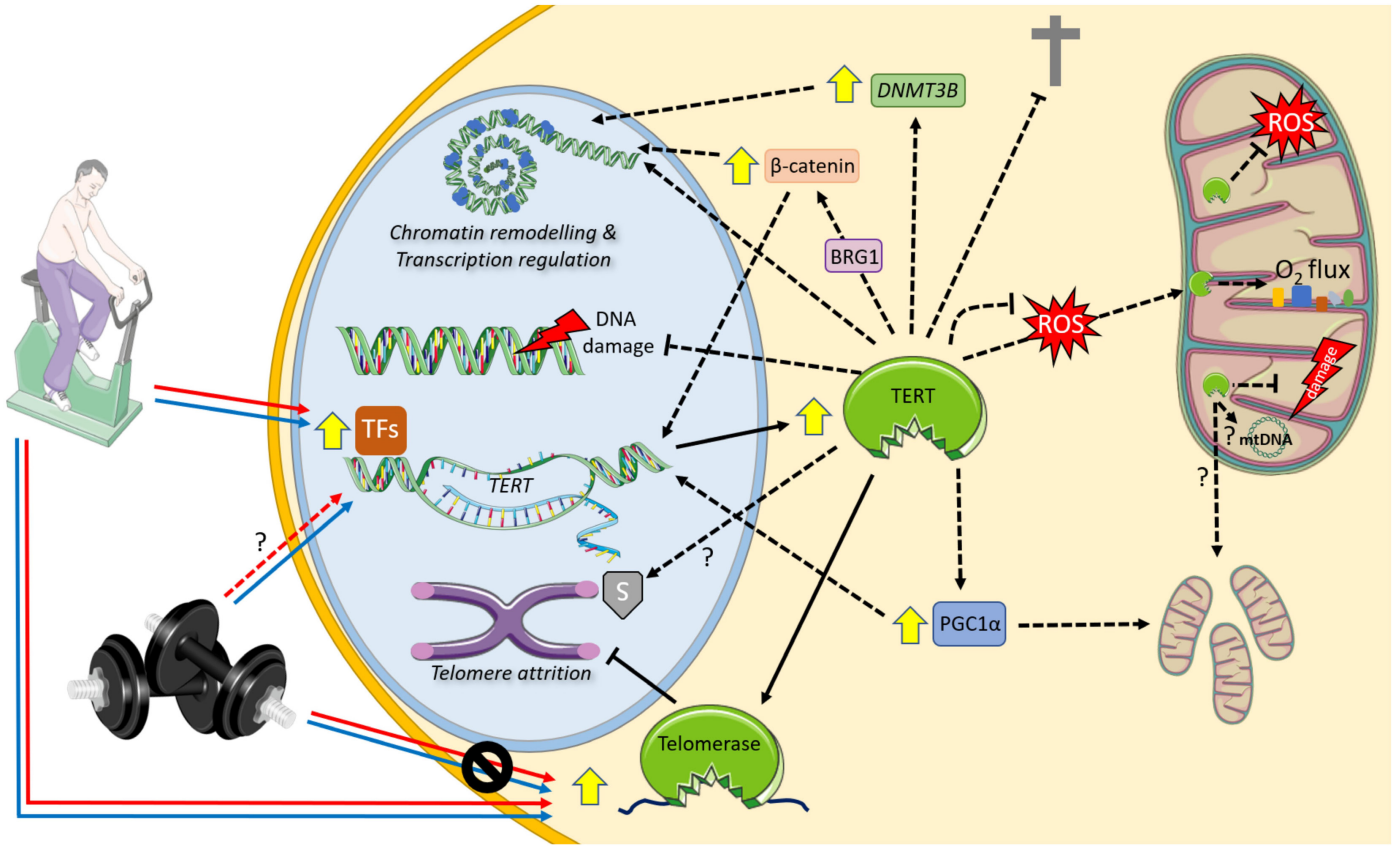
Canonical and extra‐telomeric functions of telomerase and the exercise‐mediated regulation in healthy ageing. A single bout and long‐term exercise training increases the expression of *TERT* and telomerase activity presumably to attenuate telomere attrition through its canonical function. Although long‐term resistance training appears to increase *TERT* expression in leukocytes, neither a single bout nor long‐term training modulates telomerase activity in human PBMCs. As part of its extra‐telomeric functions, TERT acts as a chromatin remodeller (through interactions with BRG1, as well as increasing *DNMT3B* expression) and influences transcriptional profiles. TERT protects against DNA damage, cell death, and safeguards the telomeres through the formation of a complex comprised of heat shock protein 70 and Apollo (Perera et al., 2019). In the presence of high reactive oxygen species (ROS), TERT is shuttled from the nucleus into the mitochondria where it prevents oxidative stress and mitochondrial DNA damage (mtDNA), enhances oxidative function, membrane potential, and transcribes RNA from the mitochondrial genome, suggesting it may promote mitochondrial biogenesis. Positive feedback loops exist between TERT and PGC1α, as well as TERT and β‐catenin/Wnt signalling. The former links TERT to metabolism and mitochondrial function, whereas the latter suggests involvement in stem cell homeostasis. Supported by Servier Medical Art. Solid lines = evidence for exercise regulation; dotted lines = evidence indicating a positive effect of TERT in other models; ? = denotes a potential role in the healthy biological ageing conferred by exercise training; red line = acute exercise effect; blue line = chronic exercise effect.

Whilst leukocyte (whole blood, subsets or peripheral blood mononuclear cell [PBMCs]) *TERT* gene expression tends to increase 1–1.5 h after a single bout of endurance exercise training in humans (Chilton et al., 2014; Cluckey et al., 2017), telomerase activity is up‐regulated immediately after an exercise session (Werner et al., 2019; Zietzer et al., 2017) and remains elevated 24 h after (Werner et al., 2019). However, whole blood leukocyte *TERT* expression was not increased 24 h after high‐intensity training in Thoroughbred racehorses (Mandal et al., 2022), suggesting that the acute exercise‐induced *TERT* expression is transient and reduces to basal levels within 24 h of training. The temporal exercise‐induced changes to TERT protein after exercise are currently unknown. The time course of TERT/telomerase changes after endurance exercise are summarised in Figure 3. One could reasonably conceive that the increased *TERT* mRNA could lead to increased TERT protein in the hour/s after a single bout of endurance exercise, which could elicit extra‐telomeric functions outside of its canonical role at the telomeres (Figure 4).

The regulating factor/s responsible for increasing telomerase activity immediately after training are currently poorly defined. Numerous transcription factors are predicted to target the TERT gene and may underpin the exercise‐induced *TERT* expression after exercise (e.g. those involved in AMPK/PGC1α/MAPK pathways (Xiong et al., 2015)). AMPK is rapidly up‐regulated immediately after exercise in human skeletal muscle, which precedes the delayed increase in PGC1α expression (1–2.5 h after exercise cessation) (Diman et al., 2016; Hawley et al., 2014). Acute exercise controls mitogen‐activated protein kinase (MAPK) signalling, as it modulates MAPK p38 and subsequently activates PGC1α (Kramer & Goodyear, 2007). Skeletal muscle PGC1α gene expression is up‐regulated in the hours following (1–4 h) a single bout of endurance and high‐intensity interval training, while PGC1α protein content is increased with long‐term training (Hawley et al., 2014). Notably, up‐regulated MAPK p38 phosphorylation was observed in plantaris and cardiac muscle of mice following a single bout of treadmill running (Ludlow et al., 2012, 2017), with concurrent changes in key shelterin genes (*Trf1/2* and *Pot1*) that regulate telomerase (Ludlow et al., 2017). Although human endurance athletes lacked detectable levels of telomerase activity and *TERT* mRNA expression in skeletal muscle (Laye et al., 2012), others have demonstrated telomerase activity and TERT protein levels are present, albeit at low levels, in healthy individuals and patients with inflammatory myopathies (Ponsot et al., 2012). Interestingly, patients with the shortest telomeres with inflammatory myopathies tended to have the highest telomerase activity and TERT expression indicating a link with inflammation (Ponsot et al., 2012). In human PBMCs, inflammation provokes TERT expression and telomerase activity, such that TNFα, increases the translocation of TERT protein from the cytosol into the nucleus and increases telomerase activity over 1–6 h via PI3K/Akt/NF‐κB signalling (Akiyama et al., 2004). Additionally, heat shock proteins (HSP) are controlled by exercise training (Henstridge et al., 2016) and physically interact with TERT (e.g. HSP70 and HSP90) (Haendeler, Hoffmann, Rahman, et al., 2003; Perera et al., 2019). Furthermore, a unique TERT/Apollo/HSP70 complex appears to protect the telomeres in neoplasms (Perera et al., 2019), which should be investigated in healthy tissues.

In spite of absent or low levels of telomerase, contractile tissue may control TERT and telomerase activity in other tissues via circulating factors (e.g. myokines or extracellular vesicle/exosome‐mediated paracrine signalling). For example, a single bout of cycling at 50% or 75% of VO_2peak_ increased acetylene co‐enzyme A carboxylase—a maker of AMPK activity—along with PGC1α and TERRA transcripts, which controls telomerase activity (Diman et al., 2016). The exercise‐induced TERRA expression facilitated by NRF1/AMPK/PGC1α signalling was speculated to regulate telomerase activity perhaps in other tissues expressing higher levels of TERT (Diman et al., 2016). This could be achieved through intercellular signalling via extracellular micro‐vesicle‐mediated transport of transcription factors, TERRA or TERT, given the exercise‐induced release of extracellular micro‐vesicles and myokines previously described (Denham & Spencer, 2020; Murphy et al., 2020). Considering exercise modulates these proteins and transcription factors, and that most are already linked to TERT in other models, future work is encouraged to examine them in context with exercise, telomere dynamics and telomerase.

Whereas a single bout of exercise causes considerable physiological challenges to meet energy and oxygen demands crucial to adaptive responses, chronic training confers systemic health and fitness benefits. Chronic exercise training increases resting levels of telomerase activity in human PBMCs and rodent tissues (Denham & Sellami, 2021) indicating a physiological adaptation to training. These findings are supported by cross‐sectional findings in young and middle‐aged endurance athletes, such that they exhibit 1.8–4.2‐fold higher leukocyte *TERT* expression (Werner et al., 2009) and PBMC telomerase activity (Hagman et al., 2020; Werner et al., 2009) compared to their inactive peers. Considering exercise training significantly preserves physical function and extends health span during chronological ageing, it is reasonable to suspect both canonical and extra‐telomeric functions of TERT may be part of the molecular mechanisms.

TERT may serve essential extra‐telomeric functions that underpin some the physiological responses to exercise training. Twenty‐one days of voluntary wheel running increased cardiac, aorta and mononuclear cell telomerase activity and reduced cell cycle and survival proteins (Chk2, p16 and p53), which were absent in second generation (G2) *Tert* ^−/−^ mice (Werner et al., 2008, 2009)—mice with intact telomeres. Endurance—running—capacity is also impaired in G4 *Tert* ^*−/−*^ mice who have considerably short and dysfunctional telomeres (Sahin et al., 2011) and physical activity abruptly decreased in 6–8 month old G5 *Terc* ^−/−^ mice within 12 h of their death (Leri et al., 2003). Both G1 and G4 *Tert* ^*−/−*^ mice exhibited reduced mitochondrial density in the heart and liver, yet mitochondria function—expression of oxidative phosphorylation genes and respiratory chain complex I/IV activity—was only impaired in G4 *Tert* ^*−/−*^ mice (Sahin et al., 2011). That mitochondrial function is only impaired in G4 compared to G1 *Tert* ^−/−^ mice could account for their poor running performance and inactivity (Sahin et al., 2011). Regular exercise training improves maximal oxygen uptake and endurance performance by eliciting cardiac adaptations (Ellison et al., 2012; Gielen et al., 2010), increasing mitochondrial content and function (Granata et al., 2018; Memme et al., 2021) and promoting skeletal muscle capillarisation (angiogenesis) (Cocks et al., 2013; Hendrickse et al., 2021; Wariyar et al., 2022). Thus, the clear telomere‐TERT‐mitochondria axis is exciting, as endurance exercise is the dominant therapy for improving mitochondrial biogenesis, structure and respiration (Gioscia‐Ryan et al., 2016; Granata et al., 2018; Memme et al., 2021). This is achieved, in part, by modulation of the dynamic regulation of the master regulator of mitochondrial biogenesis and function, PGC1α (Hawley et al., 2018; Perry & Hawley, 2018). Given the positive feedback loop between TERT and PGC1α and the essential role of PGC1α as the master regulator of metabolism, these proteins should be analysed in context with endurance training. Moreover, a single bout of endurance exercise up‐regulates skeletal muscle ERK1/2 phosphorylation in an intensity‐dependent manner via MAPK signalling (Kramer & Goodyear, 2007; Widegren et al., 1998, 2000). Exercise training also increases *VEGF* expression and angiogenesis in numerous tissues (Hoier & Hellsten, 2014; Leung et al., 2008; Morland et al., 2017). Examining TERT in context with exercise‐induced angiogenesis via eNOS/VEGF/ERK1/2 signalling pathways is also encouraged.

Although endurance training enhances skeletal muscle oxidative capacity and improves cardiorespiratory fitness, resistance training invokes conflicting signalling cascades through mTOR (Coffey & Hawley, 2017; Hawley et al., 2014; Ogasawara et al., 2019). It appears mTOR signalling directly inhibits TERT shuttling into the mitochondria reducing protection against ROS (Miwa et al., 2016), which could account for the link with telomere maintenance and improved health span after endurance exercise, rather than resistance training (Denham, O'Brien, & Charchar, 2016). However, other evidence indicates mTOR signalling can up‐regulate telomerase activity (Sundin & Hentosh, 2012), which underscores the need for future work to examine the influence of resistance training on TERT and telomerase. Currently, data on the influence of acute and long‐term resistance training on TERT/telomerase and telomere dynamics are scarce. Leukocyte *TERT* expression was increased after 12 weeks of resistance training with an emphasis on high repetitions and low load performed twice a week (Nickels et al., 2020). Conversely, telomerase activity was unaltered immediately after a bout of resistance—circuit—training and long‐term resistance training, unlike the responses observed after endurance or high‐intensity interval training (Werner et al., 2019) (Figure 3). Studying telomerase in context with resistance training regimes not placing large demand on the aerobic energy system (i.e. strength or hypertrophy) as well as concurrent training (aerobic plus resistance exercise) should be examined.

## 6 CONCLUSION

Regular exercise training is the only lifestyle factor that extends health span and preserves physical performance during ageing. Endurance training appears to attenuate telomere attrition most likely by up‐regulating TERT and telomerase activity. This, however, remains to be demonstrated in humans and will require large longitudinal studies over an extended timeframe (e.g. several years). Since its discovery in 1985, several extra‐telomeric functions of TERT have been uncovered outside its canonical role of protecting and maintaining the telomeres. These include metabolism, angiogenesis and cell survival (DNA integrity, mitochondrial function and protection from ROS). Based on the overlapping signalling pathways regulated by exercise and TERT, it is possible that the healthy biological ageing elicited by regular—lifelong—exercise may not be limited to the canonical roles of TERT at the telomeres. On the contrary, numerous extra‐telomeric functions are likely responsible and link telomere biology to other hallmarks of ageing (Lopez‐Otin et al., 2013).

Therefore, future work should seek to elucidate whether the extra‐telomeric functions presented in this review are involved in physiological adaptations to endurance training. The signalling molecules controlling *TERT* transcription and up‐regulating telomerase activity after a single bout of endurance training, as well as the time‐course of exercise‐induced changes should be confirmed. Considering the recent discovery that exercise may control alternative splicing in transgenic mice (Slusher et al., 2022), the role of *Tert* splice variants should be verified. These findings may ultimately lead to the development of novel therapeutic strategies to promote healthy biological ageing, particularly for those individuals unable to engage in or who are averse to endurance exercise. Telomere based therapies (e.g. *TERT* gene therapy) could be crucial to prolonging human health span (and lifespan) and may help address the seamless progressive burden of chronic disease and our ageing population. Given that such therapies are currently not on the horizon and are possibly decades from forming standard clinical practice, one should be encouraged to regularly engage in endurance exercise training to promote healthy biological ageing.

## AUTHOR CONTRIBUTIONS

JD drafted the manuscript, prepared the figures, reviewed and approved the final version of the work.

## FUNDING INFORMATION

This work was not financially supported.

## CONFLICT OF INTEREST STATEMENT

None declared.
